# Learning curve and performance in simulated difficult airway for the novel C-MAC^®^ video-stylet and C-MAC^®^ Macintosh video laryngoscope: A prospective randomized manikin trial

**DOI:** 10.1371/journal.pone.0242154

**Published:** 2020-11-19

**Authors:** James Pius, Ruediger R. Noppens

**Affiliations:** Department of Anesthesia & Perioperative Medicine, University of Western Ontario, LHSC- University Hospital, London, Ontario, Canada; Imam Abdulrahman Bin Faisal University College of Medicine, SAUDI ARABIA

## Abstract

Difficult airways can be managed with a range of devices, with video laryngoscopes (VLs) being the most common. The C-MAC^®^ Video-Stylet (VS; Karl-Storz Germany), a hybrid between a flexible and a rigid intubation endoscope, has been recently introduced. The aim of this study is to investigate the performance of the VS compared to a VL (C-MAC Macintosh blade, Karl-Storz Germany) with regards to the learning curve for each device and its ability to manage a simulated difficult airway manikin. This is a single-center, prospective, randomized, crossover study involving twenty-one anesthesia residents performing intubations on a Bill 1^™^ (VBM, Germany) airway manikin model. After a standardized introduction, six randomized attempts with VL and VS were performed on the manikin. This was followed by intubation in a simulated difficult airway (cervical collar and inflated tongue) with both devices in a randomized fashion. The primary end-point of this study was the total time to intubation. All continuous variables were expressed as the median [interquartile range] and analyzed using the Mann-Whitney U test. A 2-way ANOVA with Bonferroni’s *post hoc* test was used to compare both devices at each trial. All reported *p* values are two sided. The median total time to intubation on a simulated difficult airway was faster with the VS compared to VL (17 [13.5–25] sec *vs* 23 [18.5–26.5] sec, respectively; 95% CI; *P* = 0.031). Additionally, on a normal airway manikin, the VS has a comparable learning curve to the VL. In this manikin-based study, the novel VS was comparable to the VL in terms of learning curve in a normal airway. In a simulated difficult airway, the total time to intubation, though likely not clinically relevant, was faster with the VS to the VL. However, given the above findings, this study justifies further human clinical trials with the VS to see if similar benefits–faster time to intubation and similar learning curve to VL–are replicated clinically.

## Introduction

Although infrequent, difficulties with airway management remain a major cause of morbidity and mortality in anesthesia practice [1]. Video laryngoscopy has become a widely accepted method to manage patients with predicted or unanticipated difficult airways; it is also recommended by various difficult airway guidelines and societies [2–4]. Compared to direct laryngoscopy, video laryngoscopy allows for an indirect visualization of the glottis without alignment of anatomical structures. The use of video laryngoscopy is associated with an improved glottis view and a reduced number of intubation attempts in patients with a difficult airway [5]. However, multiple large-scale trials suggest that using a video laryngoscope (VL) does not automatically translate into a higher first-pass success rate [6–8]. The challenge with using a VL is not in visualizing the glottis, but rather guiding the tube through the oropharynx. After an adequate glottic view is obtained using the VL, the tube must be advanced blindly into the oropharynx before coming into view of the camera and advanced into the trachea. This creates a steep learning curve for airway trainees using a video laryngoscope for the first time [9].

The C-MAC Video Stylet (VS) is a novel device for endotracheal intubation recently introduced into practice with early clinical success [10]. The device represents a fusion of a flexible intubation endoscope and a rigid intubation endoscope. The VS consists of a rigid metal stylet with a dynamic flexible tip. The tip also contains a light source and a small video camera that can be connected to a video display. The VS is used in a similar fashion to a fiberoptic bronchoscope, with the device entering the oropharynx in a midline approach [10]. The endotracheal tube (ETT) is loaded on the stylet and is kept in place using a tube holder, allowing for advancement of the VS and tube as a single unit. The flexible tip allows for a dynamic deflection of up to 60° with the loaded tube, allowing the user to adjust the direction of the device based on observed anatomical structures. With the VS, the tube is already loaded on the device and therefore automatically brought to the glottis once an adequate view is obtained. This potentially eliminates the challenge of initially advancing the tube blindly, which could result in faster and easier tracheal intubation when compared with VL. In turn, this could present a faster learning curve with the VS.

Given the aforementioned properties of the VS, we hypothesize that learning to use the VS for endotracheal intubation on an airway manikin might be easier and result in faster intubations compared to the VL. We also hypothesize that the VS might result in faster and easier intubations compared to VL in a simulated difficult airway. We would consider a 20 sec faster time to intubation to be clinically relevant.

## Methods

### Participants

We obtained approval from the local Research Ethics Board (Western University, Project ID: 110883) and written informed consent from twenty-one participants. This study was a single-center, prospective, randomized, non-blinded, crossover study. All participants had at least 6 months clinical experience in anesthesia, extensive experience with the Macintosh blade for laryngoscopy, limited experience with video laryngoscopy (< 100 intubations using the Glidescope), and no experience with the Storz C-MAC VS and C-MAC Macintosh VL (Karl Storz, Tuttlingen, Germany).

### Study protocol

We did not perform *a priori* sample size calculation but instead used a convenience sample targeting all available anesthesia trainees. Participants were randomized to attempt tracheal intubations on a Bill 1^™^ manikin airway model (VBM, Sulz, Germany) with both devices. Before starting the study, each participant was given a standardized demonstration of both intubation devices in the form of an instructional video. This included an explanation and visual instructions on how to use both devices. The participants were then allowed to familiarize themselves with both devices. This involved anything except using the device on the airway manikin–physical manipulation of the device, assessment of video projection, loading of ETT, deflection of flexible tip, etc.

Both the VL and VS were prepared in a similar fashion before each intubation attempt. In the VL device arm, a C-MAC VL with a Macintosh size three blade was used. For all intubations in the VL device arm, a 7.5-mm internal diameter (ID) ETT (Covidien, Dublin, Ireland) was prepared and loaded onto a lubricated malleable stylet with a predefined angulation of 60°. The prepared tube was then used in conjunction with the C-MAC VL to intubate the manikin. The participant performed the intubation while one of the investigators recorded the display on the VL monitor for subsequent analysis. In the VS device arm (a detailed description of the device and its use has been described previously [10]) a 7.5-mm ID ETT was loaded onto the lubricated stylet. The VS and ETT as a single unit was then introduced into the manikin’s mouth midline and parallel to the sagittal plane. Once the distal end of the VS was observed to go past the uvula, the participant would then pull on the lever of the VS to flex the tip of the VS and bring the glottic opening into view. When adequately positioned in front of the tracheal entrance, the ETT was then advanced into the trachea, securing the airway. As the participant performed the intubation, one of the investigators recorded the image displayed on the VS monitor for subsequent analysis.

Participants were then asked to perform six intubations on a normal airway manikin with no modifications in order to attain sufficient experience with each device. This involved a total of 252 intubations (126 with each device) on the normal airway manikin. Open access software (Random.org; Randomness and Integrity Services Ltd., Dublin, Ireland) was used to randomize the sequence of attempts with each device on the normal airway manikin. Participants then attempted intubation on a simulated difficult airway with both devices in a randomized fashion. To simulate a difficult airway, a hard-cervical collar was applied to the manikin and its tongue inflated with 60 ml of air. This simulated difficult airway technique has been validated in previous studies [11, 12].

The primary endpoint of the study was the total time of intubation on a simulated difficult airway; the time from device insertion past the manikin’s lips to its removal after successful intubation. Secondary outcomes defined in advance included subjective ease of intubation on a visual analogue scale (VAS), percentage of glottic opening (POGO) achieved, first attempt success rate, and learning curves for each device on a normal airway manikin. A failed intubation attempt was defined as any that lasted over 120 seconds or where the device was removed from the manikin’s mouth and re-inserted for a second attempt. For the subjective ease of intubation on a VAS, zero was defined as very easy (e.g., no difficulties visualizing the glottic opening and/or advancing the ETT into the trachea) and ten as very difficult (e.g. multiple redirections of the device or ETT were necessary to secure the airway). For POGO, 0% was defined as none of the glottic opening was visualized (e.g., no vocal cords visible) and 100% was defined as complete visualization of the glottis from anterior commissure to the inter-arytenoid notch [13].

### Statistics

Statistical analyses were performed and graphs generated using GraphPad Prism (GraphPad Prism 6, California, USA). All continuous outcome variables were expressed as medians and interquartile ranges (IQR), and analyzed using the Mann-Whitney *U* test. The Hodges-Lehmann estimator was used to calculate the 95% confidence interval (CI) for the median difference. With regards to the learning curve in using VL and VS on a normal airway manikin, a 2-way ANOVA with Bonferroni’s *post hoc* test was used to compare both devices to determine if device type or trial number was a statistically significant variable. All reported *p-*values were two-sided and *p-*values less than 0.05 were considered statistically significant.

## Results

A total of twenty-one anesthesia trainees completed the study. Their experience in clinical anesthesia and prior experience with each device are detailed in Table 1.

**Table 1:** Characteristics and intubation experience of participants.

Parameter	*n* = 21
Anesthesia training level, years, median	2
Prior experience with Glidescope intubations	
0–25 applications	3
25–50 applications	8
50–100 applications	3
>100 applications	7
Prior experience with C-MAC VS	none

### Total time to intubation in difficult airway

**Fig 1:**
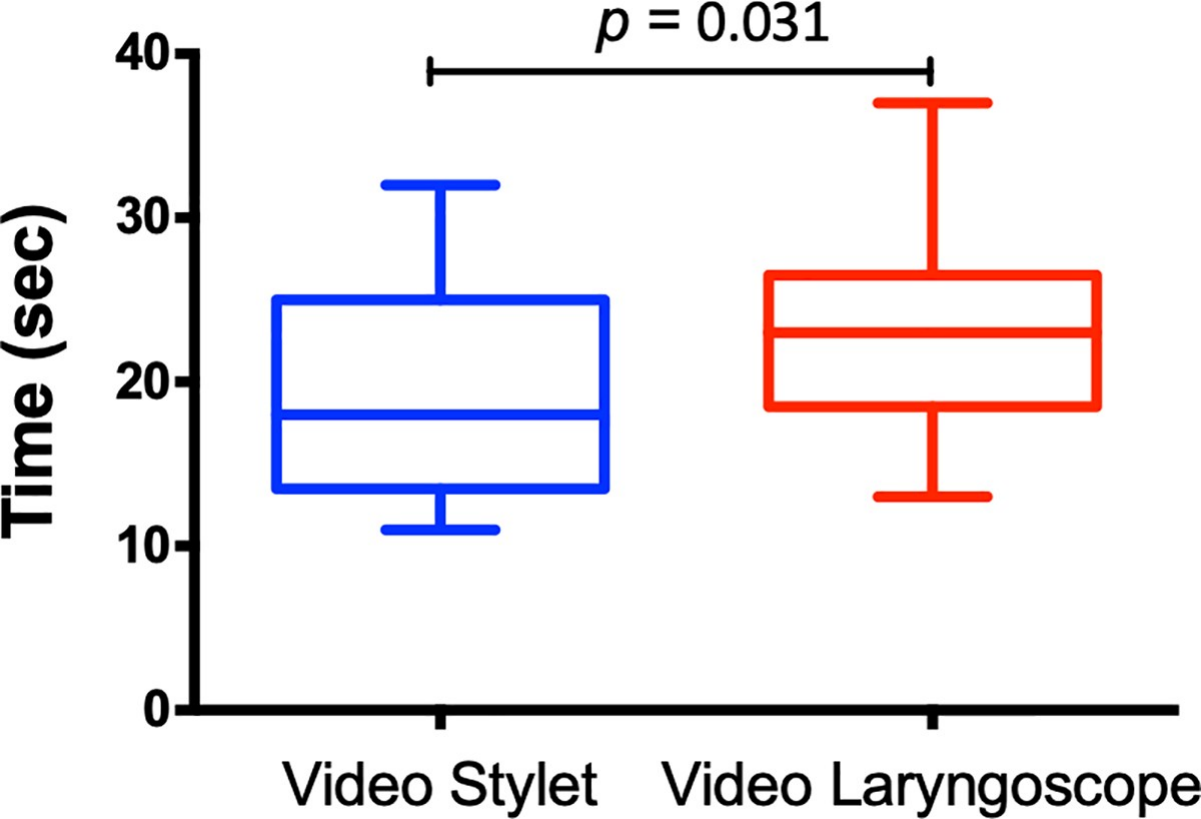
Total time to intubation in a simulated difficult airway (*n* = 21). Boxes span the interquartile range; the line within each box denotes the median, and whiskers indicate the minimum and maximum values.

The median [IQR] total time to intubation on a simulated difficult airway manikin was faster with the VS than with the VL (17 [13.5–25] sec *vs* 23 [18.5–26.5] sec, respectively, Fig 1; median difference, 6 sec; 95% CI; *P* = 0.031).

Comparison of the various stages of intubation between the devices (Table 2) showed a faster time to achieve a stable glottis view (VS 10 [6.5–12] sec *vs* VL 12 [8.5–15.5] sec, median difference, 2; 95% CI; *P* = 0.036) and to remove the device after ETT intubation (VS 1 [1–1] sec *vs* VL 2 [1–2] sec, median difference, 1; 95% CI; *P* = 0.0002) with the VS compared to the VL. The time from stable glottis view to ETT intubation was faster but not statistically significant with the VS (VS 7 [4.5–10] sec *vs* VL 8 [6–9.5] sec, median difference, 1; 95% CI; *P* = 0.31).

**Table 2:** Intubation metrics of difficult airway.

Parameter	Video Stylet *n* = 21	Video Laryngoscope *n* = 21	*P* value
Total time to intubate, sec, median [IQR]	17 [13.5–25]	23 [18.5–26.5]	0.031
Time from lips to stable glottis view, sec, median [IQR]	10 [6.5–12]	12 [8.5–15.5]	0.036
Time from stable glottis view to ETT intubation, sec, median [IQR]	7 [4.5–10]	8 [6–9.5]	0.31
Time from ETT intubation to removal of device, sec, median [IQR]	1 [1–1]	2 [1–2]	0.0002
VAS score of ease of intubation, median [IQR] {0 = easy, 10 = difficult}	2 [2–3]	4[3–5]	0.0007
1^st^ attempt success (%)	100	100	
POGO %, median [IQR]	100 [100–100]	10[10–25]	<0.0001

IQR = interquartile range; POGO = percentage of glottic opening; VAS = visual analogue scale

### Secondary endpoints

The median [IQR] VAS score indicated that the ease of intubation in a simulated difficult airway manikin was rated easier with the VS than with the VL (2 [2–3] *vs* 4 [3–5] respectively; median difference, 1; 95% CI; *P* = 0.0007) (Table 2). All tracheal intubations in both groups were successful on the first attempt. The glottic visualization (POGO%) was reduced with the VL than compared to the VS (Table 2) in the simulated difficult airway manikin.

### Learning curves

**Fig 2:**
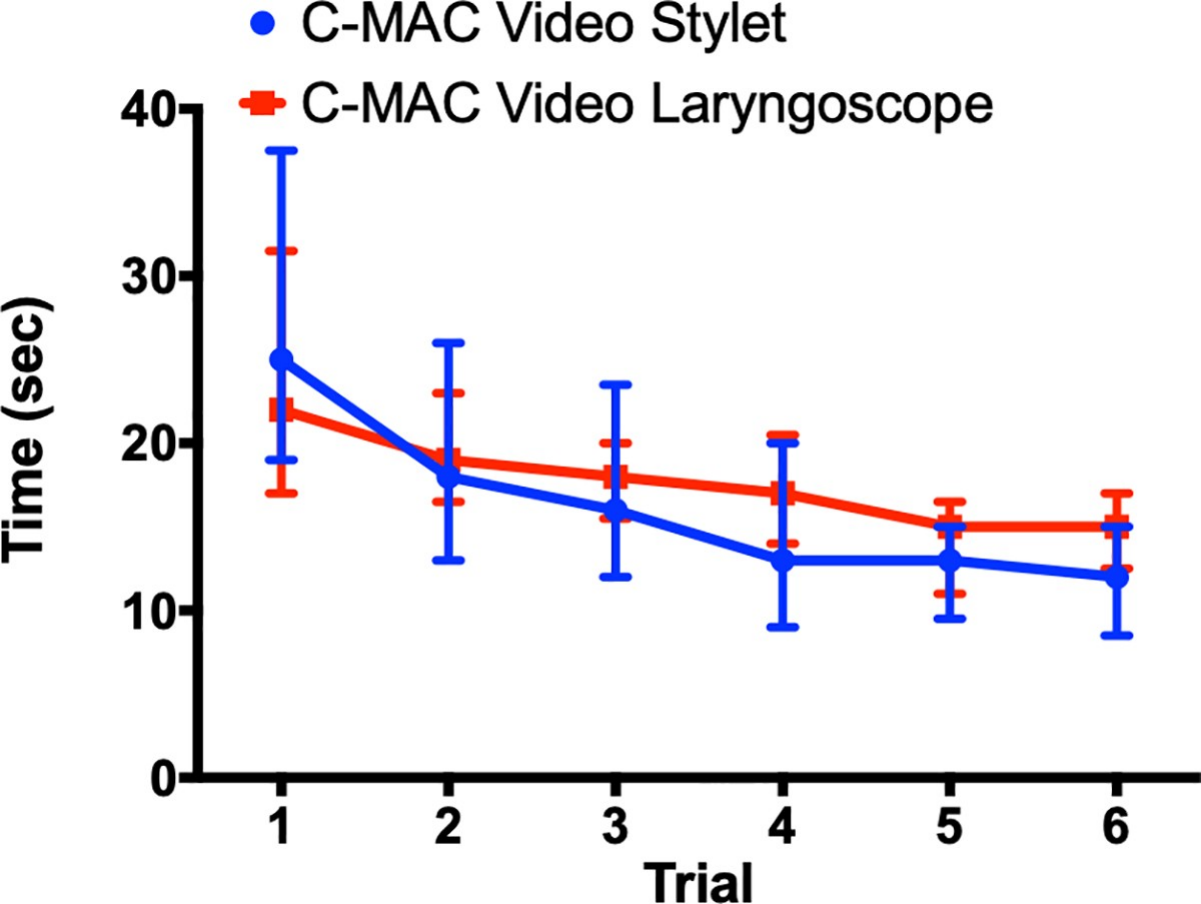
Learning curves in a normal airway (*n* = 21). Learning Curve for C-MAC Video Stylet and C-MAC Video Laryngoscope; median with 95% confidence interval. Comparison by two-way ANOVA confirmed trial number as a statistically significant interaction: device effect, *p* = 0.83; trial number effect, *p*<0.0001.

Prior to intubating the simulated difficult airway, each participant performed six intubations with each device on a normal airway manikin. This was used to generate a learning curve (Fig 2). For the first trial, the median total time to intubation was similar between devices (VS 21 [15.25–27.25] sec *vs* VL 21.5 [14–27.75] sec, *P =* 0.57). Subsequent trials also showed a similar but shorter median total time to intubation between the two devices. On the sixth trial, the total time to intubation was similar between devices (VS 12 [9.25–15.75] sec *vs* VL 14 [13–17] sec, *P* = 0.21); however, the total time to intubation was significantly shorter compared to the first trial for each corresponding device (VS 21 sec *vs* 12.0s sec *P* = 0.0028 and VL 21.5 sec *vs* 14 sec *P* = 0.027). Comparison by two-way ANOVA confirmed trial number as a statistically significant interaction (device effect: *P* = 0.83; trial number effect: *P* <0.0001).

All tracheal intubations in the normal airway manikin for both groups were successful on the first attempt. The POGO was significantly lower with the VL than compared to the VS (41.7 [39.0–60.2] % *vs* 100 [100–100] % respectively; median difference, 58.3; 95% CI; *P* = 0.002). The median [IQR] VAS score indicated that the ease of intubation was similar for both devices in a normal airway manikin (VS 1.71 [1.40–2.35] *vs* VL 2.44 [1.90–2.90]; median difference, 0.62; 95% CI; *P* = 0.10).

## Discussion

The results of this study demonstrate that intubating a simulated difficult airway manikin is faster and subjectively easier with the VS than the VL. The difficulty of the simulated airway is established by the fact that it had a longer time to intubation for both devices than the sixth and final attempt with each device in the normal airway. Additionally, on a normal airway manikin, the VS has a comparable learning curve to the VL. To the best of our knowledge, this is the first study to demonstrate the utility of the VS for tracheal intubation in a simulated difficult airway manikin and its corresponding learning curve on a normal airway.

Prior studies comparing the Bonfils–a rigid intubating endoscope similar to VS but with a rigid, fixed curved tip [14]–to the C-MAC VL have demonstrated a shorter (albeit not statistically significant) total time to intubation in a simulated difficult airway [15, 16]. For example, Kaplan et al. [16] showed a total time to intubation in a simulated difficult airway manikin (cervical collar and tongue swelling) with the Bonfils to be 14 sec compared to 15 sec with the C-MAC VL. Potential explanation for the faster total time to intubation compared to our study is likely related to the use of a different manikin (Bill 1^™^ manikin vs SimMan 3G manikin). As such, it is more useful to compare the difference in total time to intubation between the devices in each study. Kaplan et al. [16] showed a difference of 1 sec between the Bonfils and VL in favor of the former. Our study showed a difference of 6 sec between the VS and VL, with the former being faster in a simulated difficult airway. A potential explanation for the superior results seen with the VS over other rigid stylets could be the flexible tip, which allows for easier navigation of the airway. Preceding studies have shown improved success rates using rigid stylets with different curvatures [17]. An additional reason for faster and easier intubation with the VS compared to the VL is that the pathway to the cords to be navigated by the ETT have already been visualized by the VS. If the vocal cords can be visualized with the VS, an unobstructed passageway can be created to advance the ETT, but with VL, visualization of the cords does not guarantee an unobstructed path for the ETT. This potentially precipitates difficulties advancing the tube, leading to a slower intubation [18]. Analysis of various stages of intubation demonstrated a statically significant faster time for all stages with the VS than compared to the VL, with the exception of the time from stable glottis view to ETT intubation. Although this stage of the intubation was faster with the VS, it was not statistically significant, which might suggest that our study is underpowered to distinguish a difference. Although the POGO is significantly better with the VS, this fact does not necessarily translate into a faster or easier intubation. Gu et al. have demonstrated that a deliberately restricted laryngeal view (smaller POGO) is associated with a faster and easier intubation with the VL [19].

Despite participants having no prior experience with the VS, the learning curve for the VS is comparable to the VL on a normal airway manikin. This could be due to the former being less labor intensive and requiring only a minimal mouth opening and cervical flexion to obtain an adequate view. We elected to compare the VS to the VL instead of direct laryngoscopy (DL) to create our learning curve because our participants had significant (albeit variable) prior experience with DL, minimal prior experience with VL, and none with VS. This would have most likely favored DL for a faster learning curve. Previous studies with the Bonfils have demonstrated a steeper learning curve compared to DL using the Macintosh blade [15]. Since VL plays an important role in the management of the difficult airway or failed airway after DL [20], it is more prudent to assess the learning curve between VS and VL to assess any differences. Although we expected the VS to have a faster learning curve, both devices demonstrated a similar learning curve, which could be a reflection of the common initial steps taken for both DL and VL.

Given the above findings with the VS, including faster intubation of difficult airway and comparable learning curve to VL, these results warrant further human clinical trials. Using the data obtained from this study, assuming a common standard deviation of 6 seconds, an alpha error of 0.05, and a beta error of 0.1, twenty-two participants in each group are necessary to find a difference of 6 seconds in human clinical trials. Accounting for uncertainties in these assumptions, we will include a total of 60 participants (30 for each device).

There are several limitations to this study. This was a manikin study; unlike a clinical setting, the manikin anatomy is fixed and cannot mimic real physiological conditions, including secretions, bleeding, and fogging caused by body temperature. These factors can all make managing the human airway more challenging, and their absence in this study limits the interpretations that can be drawn. For example, in the clinical setting, the SensasScope–a semirigid S-shaped stylet with a flexible tip similar to the VS–has a slightly but significantly longer total time to intubation compared to the McGrath VL [21]. As such, one has to be cautious extrapolating data obtained from this manikin study to draw conclusions about the VS in a clinical setting. However, manikins have been previously used to compare different devices and have been a reliable surrogate for clinical outcomes and allow initial experience with a novel device before using it in patients [18]. Given the limited existing data on this novel device, an initial manikin study is justified to investigate the device’s feasibility and safety before proceeding with clinical trials.

The number of participants in this trial is relatively low. However, it is unlikely that the results of this trial will be altered by including more participants. Our results indicate that there might be clinically relevant differences in a clinical trial, and therefore further trials should be focusing on human patients.

There were known systemic biases that could not be avoided. The study investigators would have become aware of the assigned randomization while assessing the video recordings for ease of intubation and POGO. The investigators are also aware of the study’s hypothesis and objectives, which could have introduced bias when analyzing the recordings. These limitations are common in many airway studies [14–16].

## Conclusions

We demonstrated that using the VS resulted in a significantly shorter total time to intubation and was associated with easier intubation as measured on a VAS in a simulated challenging airway manikin. Our study suggests that the VS has a comparable learning curve to the C-MAC VL. Although we did not meet our 20 sec faster time to intubation with the VS, this study's results do warrant further evaluation of the VS clinically.

The three-step approach to characterize a novel instrument for airway management should include (1) manikin trial, (2) clinical trial focusing on success and learning curve and (3) clinical trial focusing on special circumstances (e.g. expected difficult airway). With this study, we have completed the first step, and we will be moving on to the next two steps to see if the above findings can be replicated in a real-life clinical setting.

## Supporting information

S1 Table Raw dataset for intubation of difficult airway. (XLSX)

S2 Table Raw dataset for learning curve in a normal airway. (XLSX)
